# Indium Tin Oxide Nanowires: Growth Methods, Applications and Perspectives

**DOI:** 10.3390/nano16181180

**Published:** 2026-09-18

**Authors:** Qiang Li

**Affiliations:** 1 Key Laboratory of Physical Electronics and Devices for Ministry of Education and Shaanxi Provincial Key Laboratory of Photonics & Information Technology, Xi’an Jiaotong University, Xi’an 710049, China; liqiang2014@mail.xjtu.edu.cn; 2 School of Electronic Science and Engineering, Xi’an Jiaotong University, Xi’an 710049, China

**Keywords:** indium tin oxide nanowire, vapor–liquid–solid mechanism, transparent and flexible electrode, optoelectronic device

## Abstract

Indium tin oxide (ITO) is an important transparent conductive oxide, with wide applications in the field of optoelectronics. ITO nanowires (ITO NWs) not only have high surface volume ratio and excellent electrical–thermal properties, but they also maintain the advantages of high transmittance and conductivity of ITO film. They are used to prepare the current-spreading layers of devices with special morphologies. Gas sensors and transparent flexible devices have incomparable advantages over other metal oxide nanowires at present. Comprehensive review and summary of the preparation and application of ITO NWs is helpful for exploring common points in the growth mechanisms of ITO NWs under different fabrication methods, and for matching the appropriate nanowire morphology according to the application field. This paper summarizes and compares the main academic achievements in the preparation and application of ITO NWs, and the leading characterization techniques that contribute to the advancement of ITO NWs are reviewed in detail. The different preparation methods, different morphology control methods, electrical and optical properties of ITO NWs, and their applications in optoelectronic devices, gas sensors, supercapacitors, solar cells, and other devices are systematically considered.

## 1. Introduction

With the continuous advancement of technology, nanomaterials have become an important and powerful pillar in the current development of high-tech applications. The preparation and related properties of nanomaterials are at the forefront of scientific research. Controllable, effective, cost-effective, uniform, mass-scale preparation of nanomaterials with excellent performance is one of the most challenging topics in nanotechnology. Due to their high surface-to-volume ratio and excellent electrical and thermal properties, nanowire structures have been widely applied in fields such as electronics, optoelectronics, chemical/biological sensors, energy harvesting/conversion, etc. Since the discovery of the vapor–liquid–solid (VLS) growth mechanism by Wagner and Ellis in 1964, many innovative methods have emerged, such as chemical vapor deposition, magnetron sputtering, molecular beam epitaxy, etc., which have greatly promoted the synthesis and application of nanowires [1,2,3,4,5,6,7,8,9,10,11,12].

Indium tin oxide (ITO) material exhibits excellent chemical and thermal stability, along with good adhesion to substrates and pattern etching characteristics. As a transparent conductive film, it has been widely applied in solar cells, displays, and LED lighting devices [13,14,15,16,17,18,19]. ITO nanowires (ITO NWs) not only retain the nanostructured characteristics but also maintain the high transmittance and conductivity advantages of ITO bulk materials. They possess unparalleled advantages over other metal oxide nanowires in the fabrication of special morphology devices for light emission and conductive layers, gas sensing devices, and transparent flexible devices. Due to their immense application value, ITO NWs have garnered significant interest from numerous researchers in recent years. With in-depth research, several methods for preparing ITO NWs have been developed, and it has been discovered that nanoscale ITO materials exhibit numerous excellent properties. For instance, M. Afshar et al. reported the preparation of ITO NWs using the chemical vapor deposition (CVD) method and found that these nanowires exhibit superior sensor performance [15]; Q. Wan et al. prepared ITO nanowire arrays using the physical vapor deposition (PVD) method with a catalyst and discovered their excellent field emission properties [16]; M. Z. Alam et al. pointed out that ITO NWs can achieve optical nonlinearity hundreds of times higher than other materials, indicating their potential for significant contributions in multiple photonics applications in the future [17].

**Figure 1:**
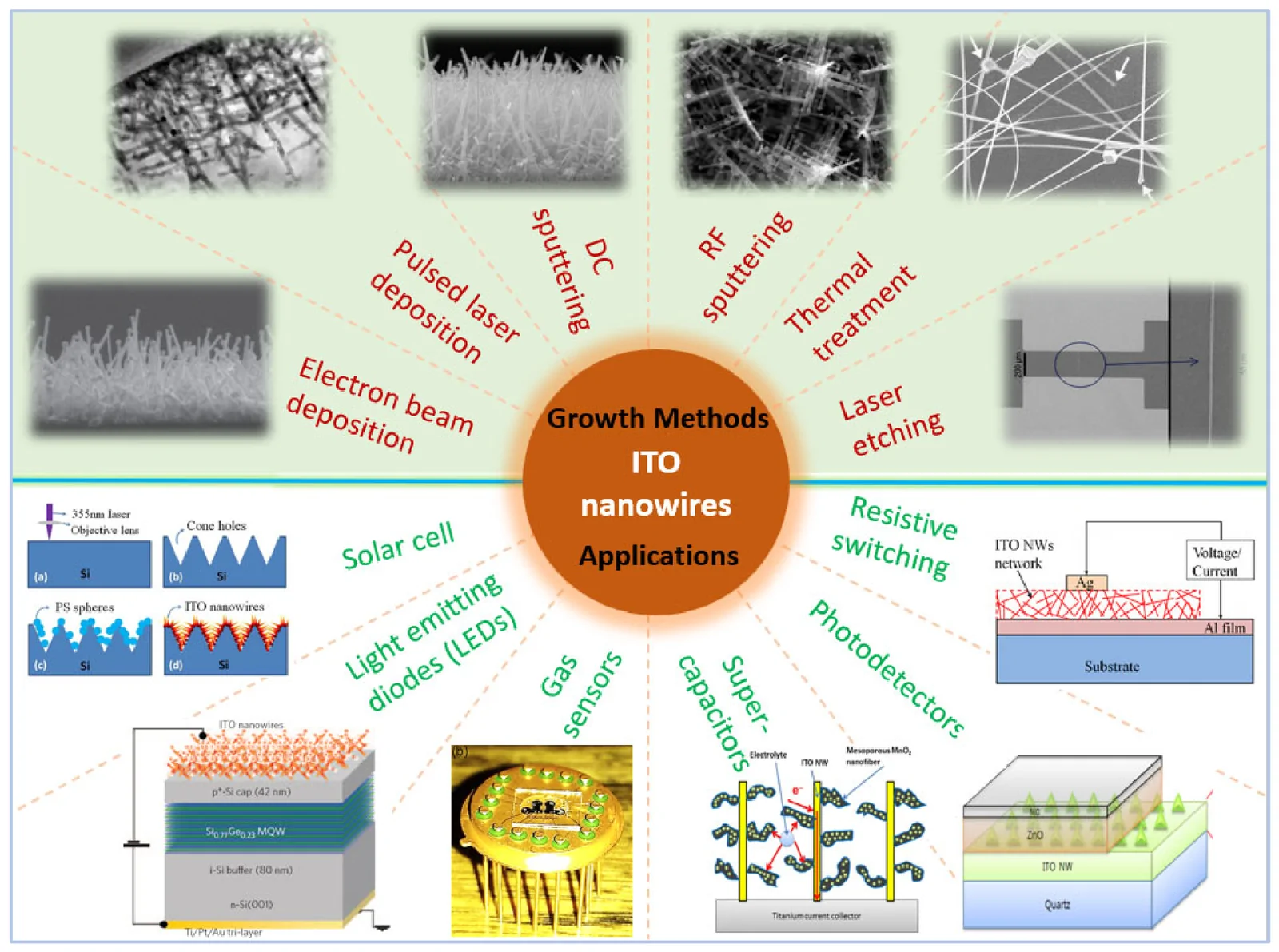
Overview of the growth methods and applications of ITO NWs.

This paper provides an overview of the current major academic achievements in the field of ITO NWs, as shown in Figure 1. It systematically reviews the synthesis mechanisms and methods of ITO NWs, including thermal treatment, magnetron sputtering, laser deposition, co-precipitation annealing, laser inscribing, and subsequent chemical etching. It compiles common morphologies of ITO nanowires, along with their corresponding preparation conditions and electro-optical properties. This article systematically summarizes the specific applications of ITO nanowires in devices such as light-emitting diodes (LEDs), gas sensors, photodetectors, supercapacitors, and solar cells.

## 2. Growth Methods of ITO Nanowires

The synthesis of ITO NWs is typically based on a VLS growth mechanism, where reactant vapors are absorbed by a catalyst until supersaturation within the liquid alloy leads to the growth of nanowire. The self-catalytic method and the gold-catalytic method have been the most widely used. Other methods, such as chemical synthesis and etching, have also been applied in recent years. The synthesis mechanisms, preparation process, and properties of nanowires (e.g., transmittance, resistivity) are systematically described in this section.

The self-catalytic VLS process avoids the introduction of other by-products, which greatly purifies the nanostructures [18]. The temperature required for self-catalytic growth is generally lower than 500 °C, but some methods use higher temperatures [19,20]. Taking 500 °C as the dividing point, we call the methods with temperature lower than 500 °C ‘low temperature methods’, and the methods with temperature higher than 500 °C ‘high temperature methods’. No matter which temperature is used for preparation, one of the biggest drawbacks of self-catalytic growth is that the diameter of nanowires cannot be controlled [21].

### 2.1. Self-Catalytic VLS Growth at Low Temperatures

#### 2.1.1. Electron Beam Deposition

Compared with chemical vapor deposition (CVD) and pulsed laser deposition (PLD) method, electron beam (e-beam) deposition is the most commonly used and economical method for growing large-area uniform ITO NWs at low temperatures [8].

In the work of R. Rakesh Kumar et al. [6,8], ITO NWs were synthesized over a large area of glass and silicon substrates by electron beam evaporation at low substrate temperatures of 150–400 °C without using additional reactive oxygen gas and metal catalyst particles. ITO target materials of In_2_O_3_:SnO_2_ (90:10, wt%) were used for the ITO NWs’ growth. The growth processes were carried out at 1 × 10^−5^ mbar for 15 min with a deposition rate of ~2.5 Å/s. The micrometer-long ITO NWs were grown at 300–400 °C. The structural studies of self-catalytic nanowire growth conducted by transmission electron microscope (TEM) showed high crystallinity and good crystal quality. The effects of growth duration, evaporation rate, oxygen partial pressure, and substrate type on the nanowire growth were also considered in that work.

**Figure 2:**
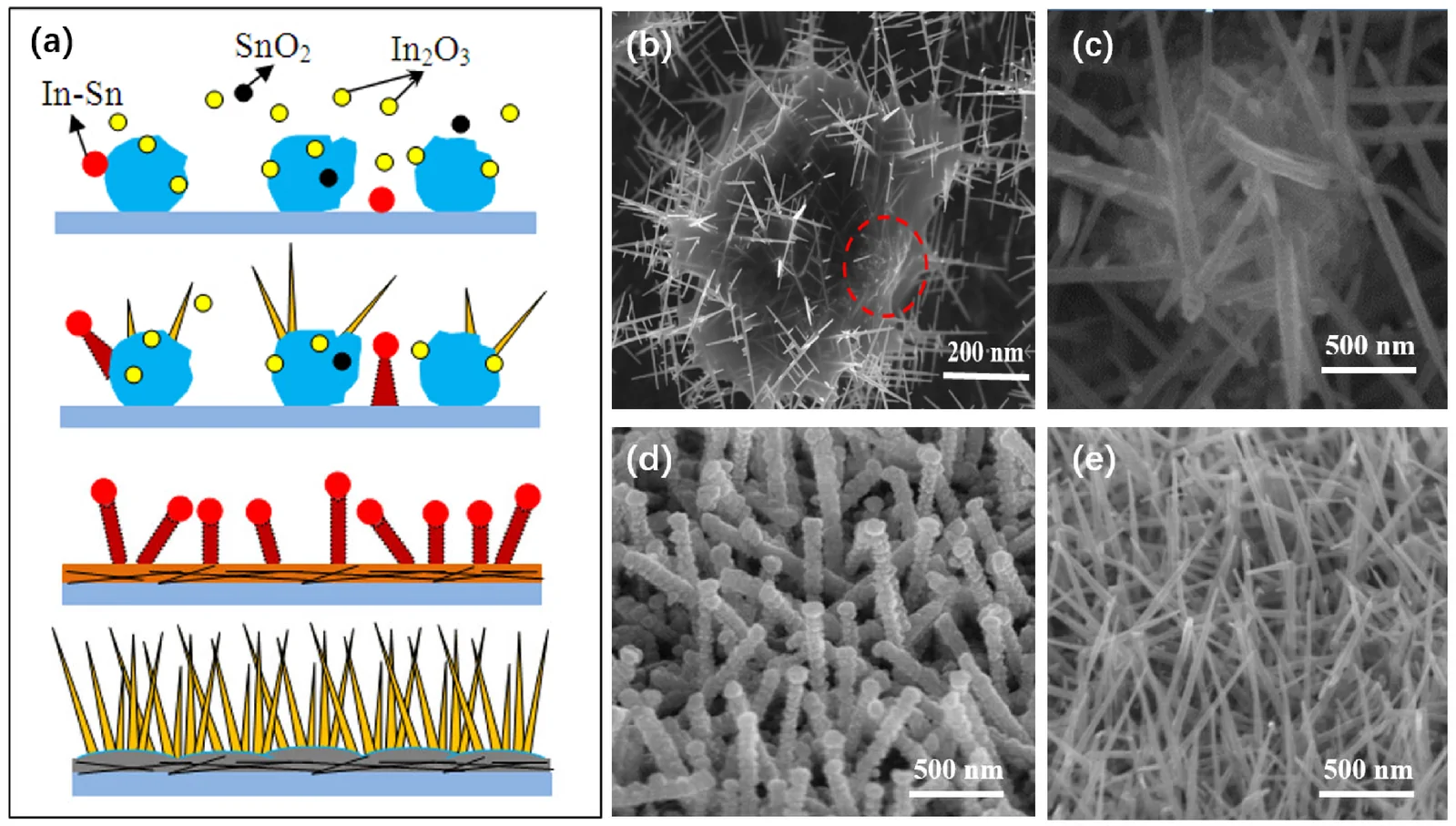
(**a**) The ITO NWs growth process via PS spheres. (**b**) ITO NWs in the early growth stage, and the NWs just begun to grow in the red dashed circular. (**c**) ITO NW networks. (**d**) The ITO nanorods with head particles. (**e**) ITO NWs.

Q. Li et al. used polystyrene (PS) as assisted material to grow high-quality ITO NWs with controllable size and density at 300 °C via an electron-beam deposition method, shown as Figure 2 [20,22]. PS spheres of 670 nm were self-assembled onto the substrate surface. An ITO source (In_2_O_3_:SnO_2_ = 90:10, wt%) was then deposited on the template by e-beam at a deposition rate of 0.1 nm/s for 15 min, with the chamber temperature stabilizing at 300 °C and pressure less than 5 × 10^−4^ Pa. Annealing served as the final step to remove residual PS. The indium–tin (In-Sn) alloy droplets were used as nucleation sites in the melted PS spheres, and nucleation occurred at the droplet/PS interface, resulting in the ITO NWs’ growth (Figure 2a). Different temperatures (100 °C, 200 °C, and 300 °C) between the In-Sn alloy’s formation point and the PS melting point were selected. Large ITO particles formed at 100 °C (Figure 2b,c), and ITO nanorods with ball-shaped heads began to grow (Figure 2d) under 200 °C. At 300 °C, needle-shaped NWs grew (Figure 2e). The optical and electrical properties of the nanowires were characterized, resulting in an ITO NWs network with low reflectivity and low sheet resistance.

Hak Ki Yu and Jong-Lam Lee developed a method using ion beam-assisted deposition (IBAD) to control the density and alignment of ITO NWs [10]. The tin-doped indium oxide (10%) target was used to fabricate the ITO NWs on a Si substrate with a thermal oxide film of 150 nm. The growth rate was 0.5 nm s^−1^ under chamber pressure of 10^−5^ Torr and a substrate temperature of 300 °C. In the IBAD process, N_2_ was introduced into the chamber, and the gas pressure was kept at 0.2 mTorr. The emission current was set as 0.2 mA cm^−2^ with changing ion beam voltage from 0 to 150 V. The branch-type ITO NWs gradually disappeared with the increase in the ion beam voltage. The randomly distributed orientation changed to a relatively strong (222) diffraction peak. The ion beam was able to affect the density of the nanowires because the nucleation of In (Sn) nanodots was suppressed by IBAD.

#### 2.1.2. Pulsed Laser Deposition (PLD)

Thian-Khok Yong et al. grew ITO NWs on catalyst-free amorphous glass substrates at 250 °C in ambient argon (Ar) and helium (He), using a Nd:YAG pulsed laser deposition technique [23]. The wavelength for the Nd:YAG pulsed laser (EKSPLA, NL301, Vilnius, Lithuania) was 355 nm, and a 2-inch ITO target (In_2_O_3_:SnO_2_ = 90:10, wt%, Target Materials, Inc., Clinton, NY, USA) was used. The deposition rate was about 0.5 nm/s and the film thickness increased to 200 nm. The results showed that all of the ITO NWs had a crystalline structure. The ITO NWs were perpendicular to the substrate, with a diameter between 20–30 nm and a length of 700–800 nm.

#### 2.1.3. Direct Current (DC) Sputtering

**Figure 3:**
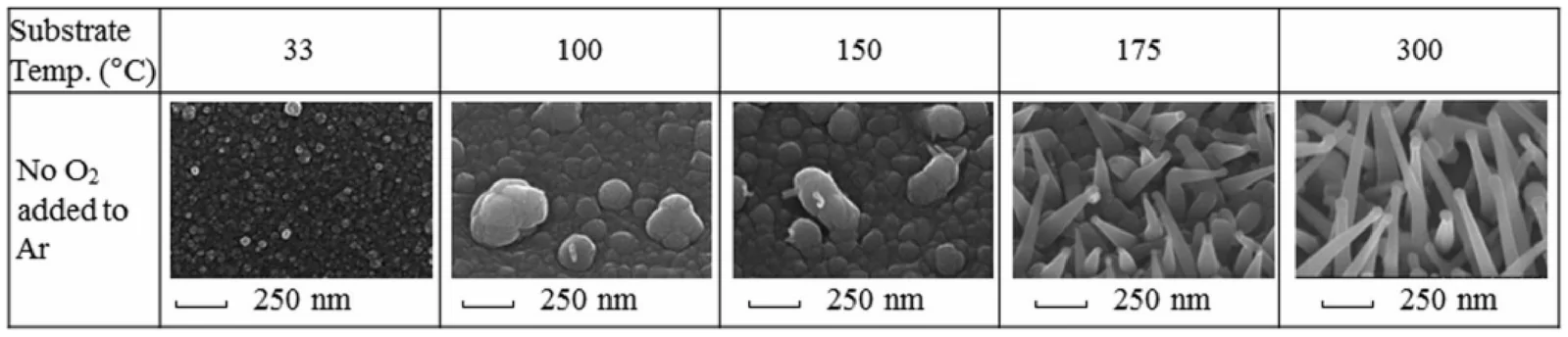
The morphology of ITO nanostructures sputtered for 600 s under different temperature substrates.

Naoki Yamamoto et al. grew ITO NWs in oxygen-free Ar sputtering gas directly after the formation of ITO films with thicknesses of 10–50 nm, using conventional magnetron sputtering [7]. A 4-inch target was selected, and the substrate was 0.7 mm thick Corning Eagle XG glass with 100 mm × 100 mm. DC power was set to 300 W and the sputtering gas pressure was 0.666 Pa. The effects of substrate temperature, deposition time, and target composition on the morphology of ITO NWs were discussed. The results were presented in Figure 3. The samples were deposited in Ar without O_2_ addition using an ITO target with 7.0 wt% SnO_2_. According to field-emission scanning electron microscopy (FESEM), the ITO NWs with trunks and ball-shaped heads were formed by a VLS mechanism via In or In-Sn droplets at 175 °C and above, because this temperature was higher than the melting point of indium (156.6 °C). Furthermore, a small amount of O_2_ partial pressure of 0.51% to 0.73% was able to suppress the growth of the NWs.

**Figure 4:**
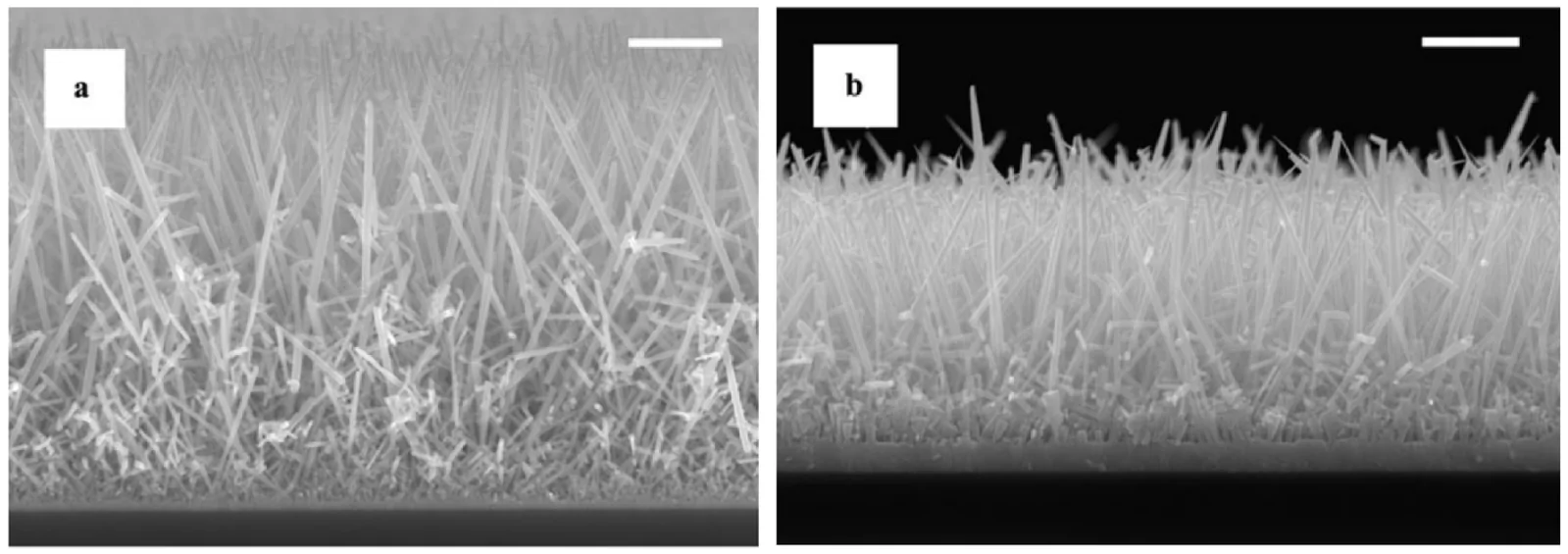
SEM images (side view) of ITO nanowires on (**a**) glass, (**b**) ITO glass. The scale bars are 1 µm.

M.K. Fung, et al. also fabricated ITO NWs by DC sputtering on different substrates without the use of catalysts or oblique deposition [3]. As shown in Figure 4, the length of the nanowires reached several micrometers, with a diameter ranging from 50 nm to 100 nm, and the nanowires possessed numerous side branches.

#### 2.1.4. Radio Frequency (RF) Sputtering

Jae Hyoung Park et al. synthesized straight ITO NWs without any side branches over 2-inch wafers via a RF magnetron sputtering method [24]. The sputtering process was conducted with a background pressure of 2 × 10^−6^ Torr, working pressure of 7.8 × 10^−3^ Torr, and RF power of 30 W in an Ar atmosphere. In metal disks with diameter of 3 mm were used as a catalyst for the growth of ITO NWs. The sputter deposition process was performed for 1 h at different substrate temperatures of 25, 100, 200, 300, 400, and 500 °C, respectively. The nucleation process of ITO NWs at 500 °C was investigated under various growth times. ITO nanorod arrays were synthesized on various substrates such as Si, glass, and ITO thin-film coated glass substrate at 500 °C for 1 h.

Q. Li et.al successfully fabricated ITO NWs by using an RF sputtering technique with a high RF power of 250 W [25]. The fabrication of the ITO NWs was optimized through the study of oxygen flow rates, temperatures, and RF power. The oxygen flow rate was 0.2 sccm and the temperature was 500 °C. The mechanism of the difference in the morphologies of ITO NWs prepared with new and used targets under the same conditions was analyzed. Nanorods were grown on the In particles as nucleation points, but NWs with smaller diameters were formed from In–Sn alloy. There were lots of oxygen vacancies in the ITO NWs, and it was also found that air voids existed inside the hollow structures of the NWs during the process of growth.

### 2.2. Self-Catalytic VLS Growth at High Temperatures

#### 2.2.1. Thermal Treatment of In and SnO Powders

When SnO and In precursors were heated to a high temperature, the following chemical reactions occurred [26,27,28]:

2SnO (g) → Sn (l) + SnO_2_ (s) (1)

SnO_2_ (s) → SnO(g) + 1/2 O_2_ (g). (2)

ITO NWs were grown based on these processes. Firstly, lots of nano Sn-In alloy droplets formed during the evaporation of precursors via a self-catalyst effect. Meanwhile, In_2_O_3_, SnO, and O_2_ were also evaporated out of the precursors and combined with Sn-In alloy droplets to form In-Sn-O ternary alloy droplets. Secondly, when the oxygen concentration in the In-Sn-O alloy droplets reached supersaturation, the ITO nanocrystals precipitated out from the alloy droplets. Finally, the ITO NWs grew based on these nanocrystals.

**Figure 5:**
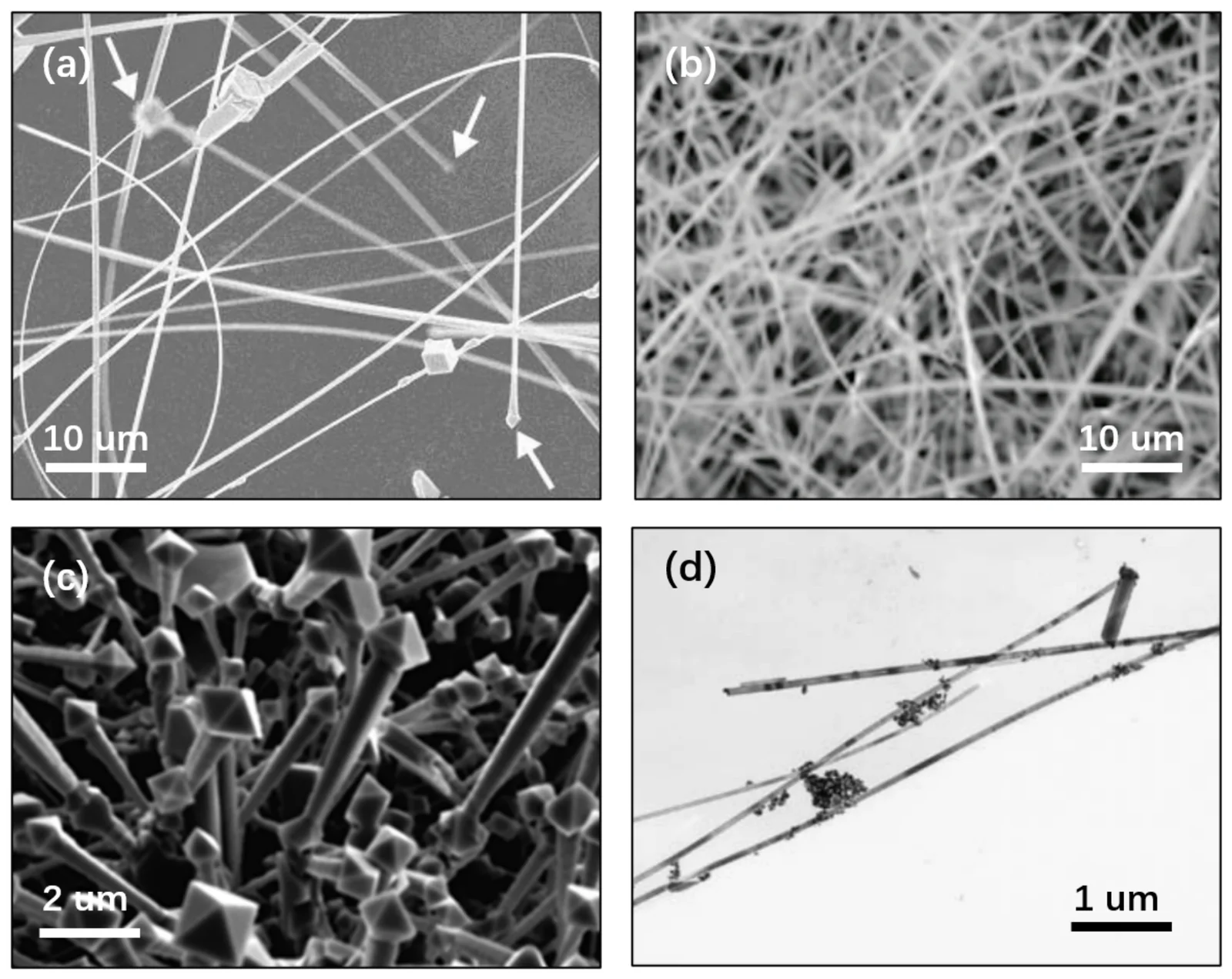
The SEM image of ITO NWs fabricated by (**a**) thermal treatment of In and SnO powders (The arrows indicate indium tin alloy.), (**b**) thermal treatment of SnO powder and InN nanowire, (**c**) thermal treatment of InN and SnO_2_ powders, and (**d**) coprecipitation and post-annealing.

Y Q Chen et al. synthesized ITO by directly heating In and SnO powders without any catalysts, dominated by a self-catalytic VLS growth mechanism [28]. The In and SnO powers were mixed (In:SnO = 90:10 wt%) and put into an alumina boat, which was inserted into a one-end-sealed quartz tube with the radius of 2 cm and the length of 20 cm. The quartz tube was then loaded into the center of the alumina tube in the furnace. Afterward, the furnace was heated up to 920 °C and kept at this heat for 20 min. The SEM image (Figure 5a) shows that the diameters of the obtained NWs were between 100 nm and 200 nm, and the lengths reached hundreds of micrometers. The HRTEM image confirms that the as-synthesized nanowire had a single crystalline structure and reveals clearly that the nanocrystal fringe spacing in the direction of (211) was 0.413 nm, corresponding to the value of (211) planes of In_2_O_3_.

#### 2.2.2. Thermal Treatment of SnO Powder and InN Nanowires

Haibo Dong et al. developed the one-step thermal evaporation–deposition method to fabricate ITO NWs at a temperature of 600 °C [1]. SnO (99.9% purity) powder and InN nanowires with a molar ratio of 10:1 were used as precursors. The powder was mixed and milled for 30 min in order to make the mixture more homogeneous. The quartz tube was heated to 600 °C, and then a quartz boat with about 0.3 g of the mixed powder was sent into the center of the quartz tube. Silicon slices, as well as quartz slices, used as the substrates, were placed downstream of the quartz boat. Then, the tube was sealed immediately and pumped down to about 100 Torr. Meanwhile, Ar was introduced as a carried gas at 50 sccm, along with oxygen. The evaporation temperature was maintained for 1 h, and then, the silicon slices were taken out directly and cooled down to room temperature. The morphology of the ITO NWs is shown in Figure 5b. The In:Sn atomic ratio was calculated to be approximately 11:1 based on the EDS and TEM results, indicating that the nanowire was a single crystal growing along the (200) direction without other impurities.

#### 2.2.3. Thermal Treatment of InN and SnO_2_ Powders

In D. Maestre et al.’s work, a mixture of InN and SnO_2_ powders (InN:SnO_2_ = 10:1, wt%) were used as the precursor. The pellets were heat-treated in an argon atmosphere. The first step involved conducting the process at 350 °C for 3 h to facilitate the desorption of N from InN. The second step involved treatment at 650 °C or 750 °C for 20 h to control the growth of nanostructures. Figure 5c shows the different nanostructures after the second step of annealing (650 °C, 20 h). The diameter of the ITO NWs varied from 80 nm to 300 nm, and it was evident that polyhedral beads were distributed along the nanowires. The results revealed complete oxidation of the In resulting from the decomposition of InN into In_2_O_3_ for all the samples [29].

#### 2.2.4. Coprecipitation and Post-Annealing

In Dabin Yu et al.’s work [30], nanocrystalline ITO with a corundum structure was synthesized via a controlled coprecipitating and post-annealing method. The InCl_3_·4H_2_O was mixed with ether in a volume ratio of 1:9, and the mixture was then transferred into an autoclave. A few drops of tin tetrachloride (SnCl_4_) were added into the autoclave and stirred vigorously. After that, 1 mL deionized H_2_O was added and stirred. The autoclave was heated at 220 °C for 24–36 h, then cooled to room temperature. Then, the co-precipitates were washed several times with absolute ethanol and distilled water, respectively, and then dried at 110 °C. Finally, the co-precipitates were loaded in a crucible and annealed at 500 °C for 2 h under ambient pressure. Figure 5d shows the SEM and TEM images of the ITO NWs. The diameter of the nanowires was between 50–70 nm, and their length reached up to 10 μm. In the synthesis process of co-precipitates, the length of the ITO NWs increased with the increase in heat treatment time; when the time exceeded 40 h, due to the completion of the hydrolysis process of In^3+^ and Sn^4+^, their length did not increase significantly.

### 2.3. Gold (Au) Catalyst Assisted VLS Growth

The diameter and length of ITO NWs have a determining effect on their optoelectronic properties [31,32]. The Au-catalyzed VLS growth method can effectively regulate the diameters and directions of nanowires. Almost all the preparation of ITO nanowires with good directionality adopts gold catalysis [33].

#### 2.3.1. Au Catalyst-Assisted VLS Growth with In, Sn, and O_2_ via Thermal Evaporation Methods

**Figure 6:**
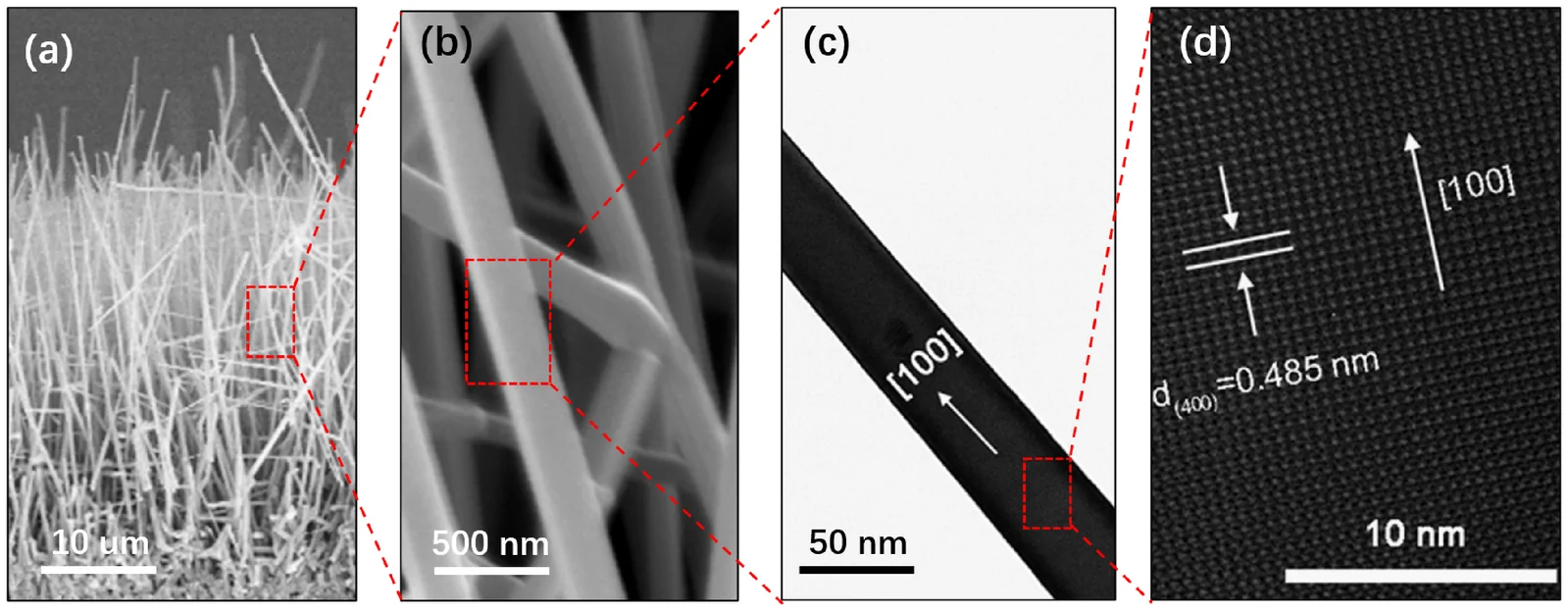
(**a**) Cross-sectional SEM image, (**b**) local enlargement, (**c**) TEM image, and (**d**) HRTEM image of ITO NW.

In gold-catalytic VLS growth, the size and spatial position of nanowires can be controlled by adjusting the size and spatial position of the gold catalyst. According to Luping Li et al.’s work [9], the range of oxygen partial pressure is crucial for the growth of the ITO NWs. They synthesized ITO NWs via the thermal evaporation method, and they considered the influence of temperature, time, oxygen partial pressure, and Au nanocluster diameters on nanowire diameter, length, and microstructure. Nanowires were grown using a mixture of In and Sn powders in an O_2_ and Ar atmosphere (0.5% O_2_ in Ar). The results (Figure 6) showed that the ITO NWs grew along the [100] direction. The length of ITO NWs could reach centimeter, and the optimal growth temperature was 750 °C [34,35,36]. M. Zervos et al. also grew ITO NWs on 1 nm Au/Si(001) substrate via the reaction of In and Sn with O_2_ at 800 °C and 10^−1^ mbar. The diameter of the ITO NWs was around 50 nm, and their length could reach up to 100 μm.

#### 2.3.2. Au Catalyst-Assisted VLS Growth with In_2_O_3_, SnO, and Graphite via Carbo-Thermal Reduction Method

**Figure 7:**
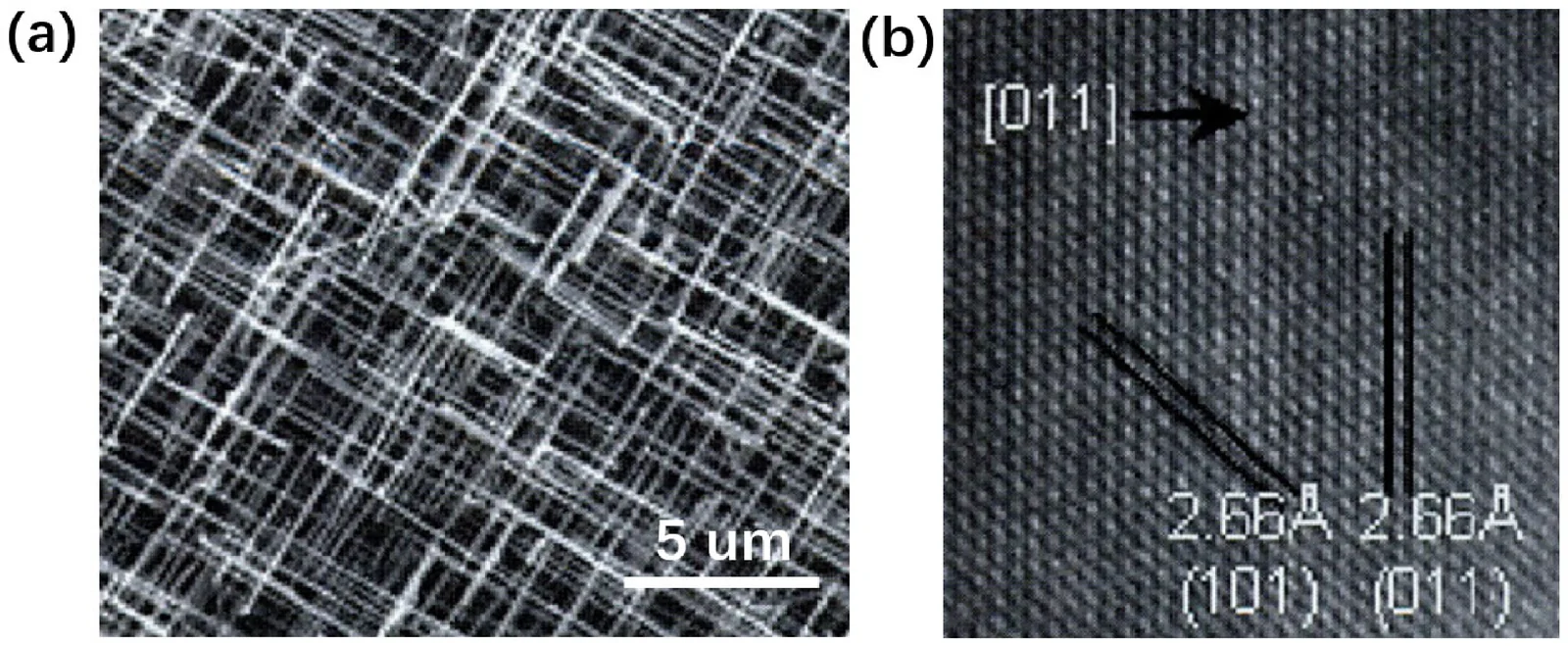
Structural characterization of ITO NWs. (**a**) Top-view SEM images, (**b**) HRTEM image showing a [011] growth direction.

Pho Nguyen et al. conducted a study varying the growth temperature and source to find their influence on the ITO NW parameters [35]. The dependence of morphology on temperature was studied first by fixing the source mixture ratio at 1:1:2 (In_2_O_3_:SnO:graphite) and varying the reaction temperature on m-sapphire substrates grown at 820 °C, 850 °C, and 880 °C, respectively. Figure 7 shows the ITO NWs with a diameter distribution of ∼81 ± 64 nm on the [011] growth direction fabricated at 850 °C.

#### 2.3.3. Au-Assisted VLS Growth of ITO NWs by Electron-Beam Evaporation

R. Rakesh Kumar et al. [36] investigated the growth behavior of Au-assisted VLS growth of ITO NWs via electron-beam evaporation (EBE) with various deposition parameters. According to their previous report, 400 °C, the optimum growth temperature for Au-catalyzed ITO NWs was used in their experiment. An ITO source (In_2_O_3_:SnO_2_ = 90:10, wt%) was set up in a graphite crucible for EBE, and a 3 nm Au film/Si substrate was used. The growth temperature was maintained at 400 °C and above. The effect of different evaporation rates (2–2.5, ∼4–4.5, and ∼6–6.5 Å/sec without external O_2_) on nanowire growth was investigated. The evaporation rate was different, and the growth mechanism could be switched between Au-catalyzed and self-catalyzed VLS mechanisms. The majority of the Au-catalyzed NWs had larger diameters while the majority of self-catalyzed NWs had only smaller diameters. The best sample was produced with an evaporation rate of ∼2–2.5 Å/sec at 400 °C under no external O_2_.

### 2.4. Others Metods

Chen et al. describe that the bottom-up and the top-down method are two elementally different approaches for NW fabrication [37]. All of the NWs we have described so far are based on the bottom-up approach. Maziar Afshar et al. used a top-down approach, where a single NW could be obtained through femtosecond laser processing and subsequent etching steps.

**Figure 8:**
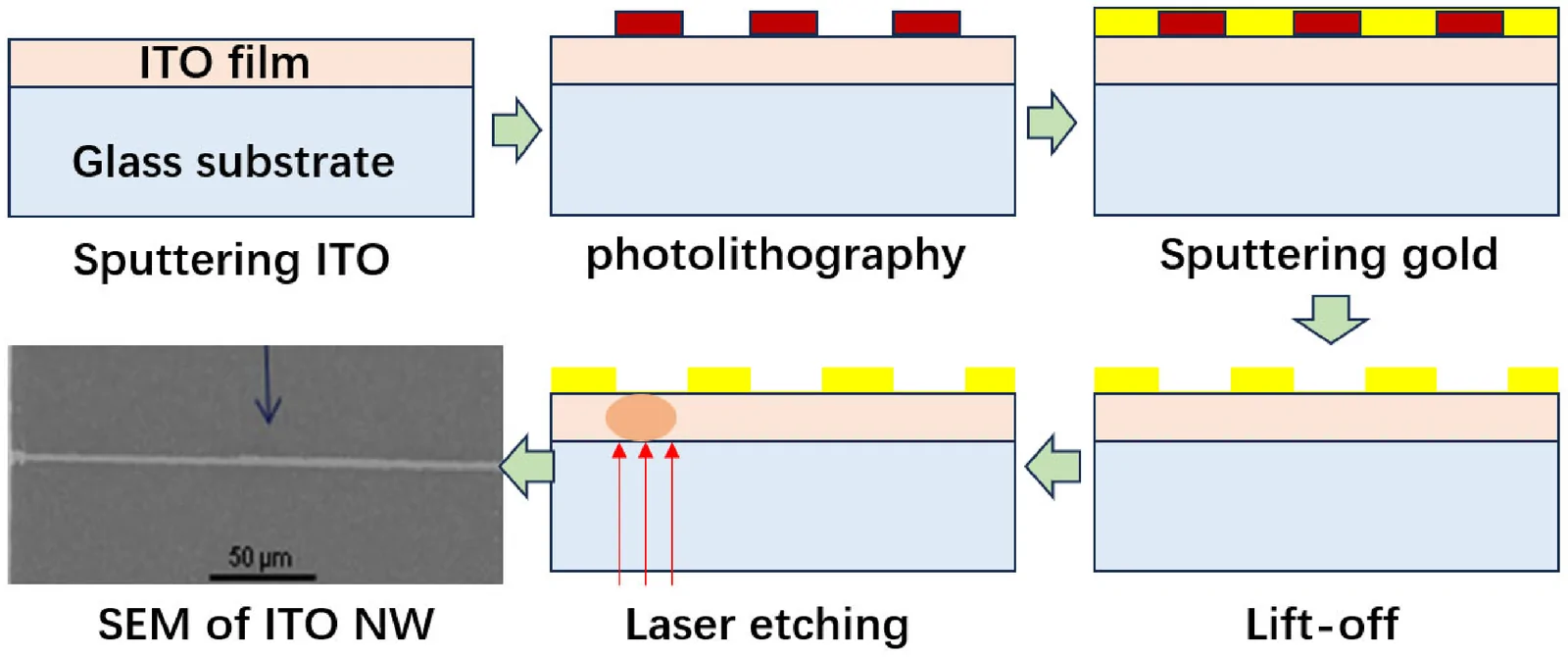
Schematic diagram of the main fabrication process for ITO NWs.

The fabrication process in shown in Figure 8. Firstly, ITO films with a thickness of 125 ± 25 nm were deposited on glass substrates via DC-sputtering. Secondly, the reversal photoresist was coated on the ITO films, and an image mask was used for photolithography processes. Subsequently, gold film was sputtered and the sample was then put in an acetone ultrasonic bath to remove the photoresist and to lift off the excess gold layer. Finally, the femtosecond laser beam was focused into the sample for writing patterns. After a series of etching processes, a single nanowire with regular morphology was obtained. Nanowires with a length of 200 μm and width of 700 nm were fabricated.

### 2.5. The Characteristics of ITO NWs with Different Morphologies

Controlling the morphology of nanowires in bottom-up synthesis and assembling them on planar substrates is important for device applications in electronics, optics, gas sensors, and energy conversion devices [38]. The growth morphology of ITO nanowires can take the following three forms: first, one-dimensional (1D) directional growth; second, two-dimensional (2D) branch growth, the formation of a nanowire network; third, three-dimensional (3D) growth. Each growth morphology needs to be achieved on the basis of the preparation method to regulate the parameters.

**Figure 9:**
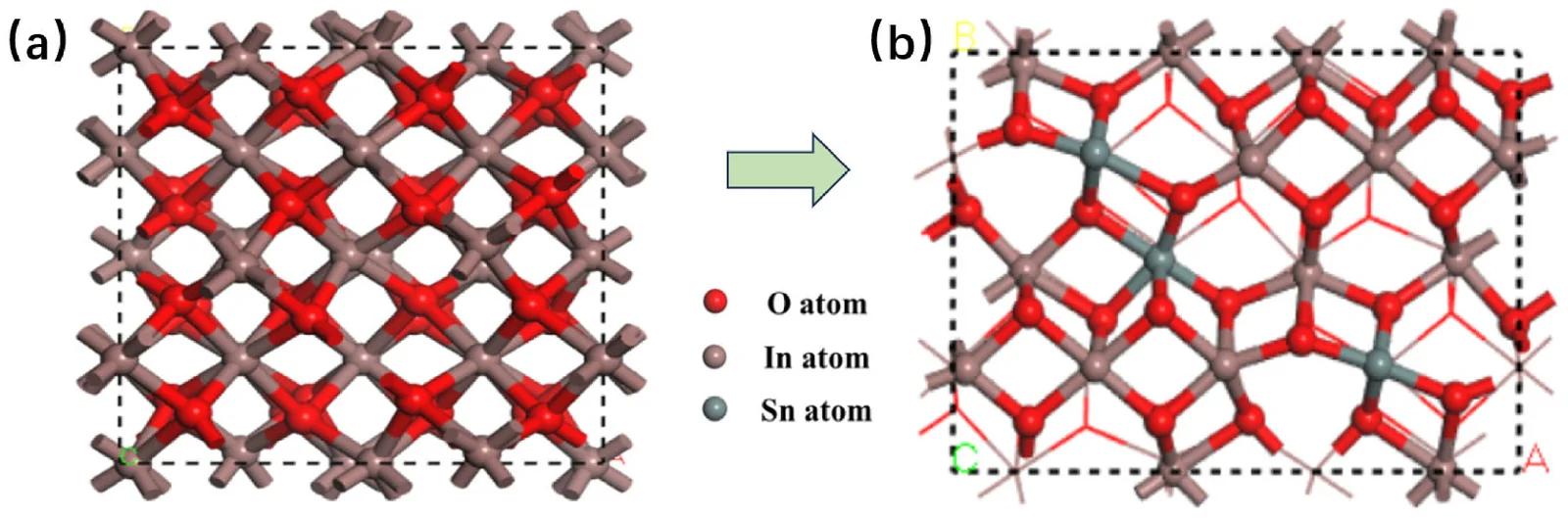
The (**a**) In_2_O_3_, (**b**) ITO cell structure established by DFT.

Nowadays, with the rise and application of artificial intelligence (AI), density functional theory (DFT) calculations can be used to design ITO nanostructures (Figure 9), and the specific properties of ITO nanostructures can be explained from the changes in crystals [39,40]. For example, different oxygen dimers have different adsorption characteristics for C_2_H_2_ gas [41], and the gas sensitivity varies with different Sn doping ratios of ITO [42]. The optoelectronic properties of these newly designed ITO nanostructures were theoretically analyzed, and then, corresponding materials were experimentally prepared. This theoretical approach is providing a new path for the preparation of ITO NWs with different shapes and functions.

Based on the above introduction to the preparation methods of ITO nanowires, we summarize them in Table 1, showing the preparation temperature, length, diameter, and the photoelectric characteristics of nanowires.

## 3. Applications of ITO Nanowires

ITO is a material of great economic significance, and ITO NWs are important morphological structures in ITO materials that exhibit novel properties. With the gradual expansion of the ITO market, ITO NWs will also play an increasingly important role. Due to its high transparency and good conductivity, ITO has played a significant role in fields such as displays, transparent memristors, and solar cells, and is expected to reach a market size of USD 300 billion by 2030. It is necessary to develop the practical methods for ITO NWs in this expanding market [62,63].

**Figure 10:**
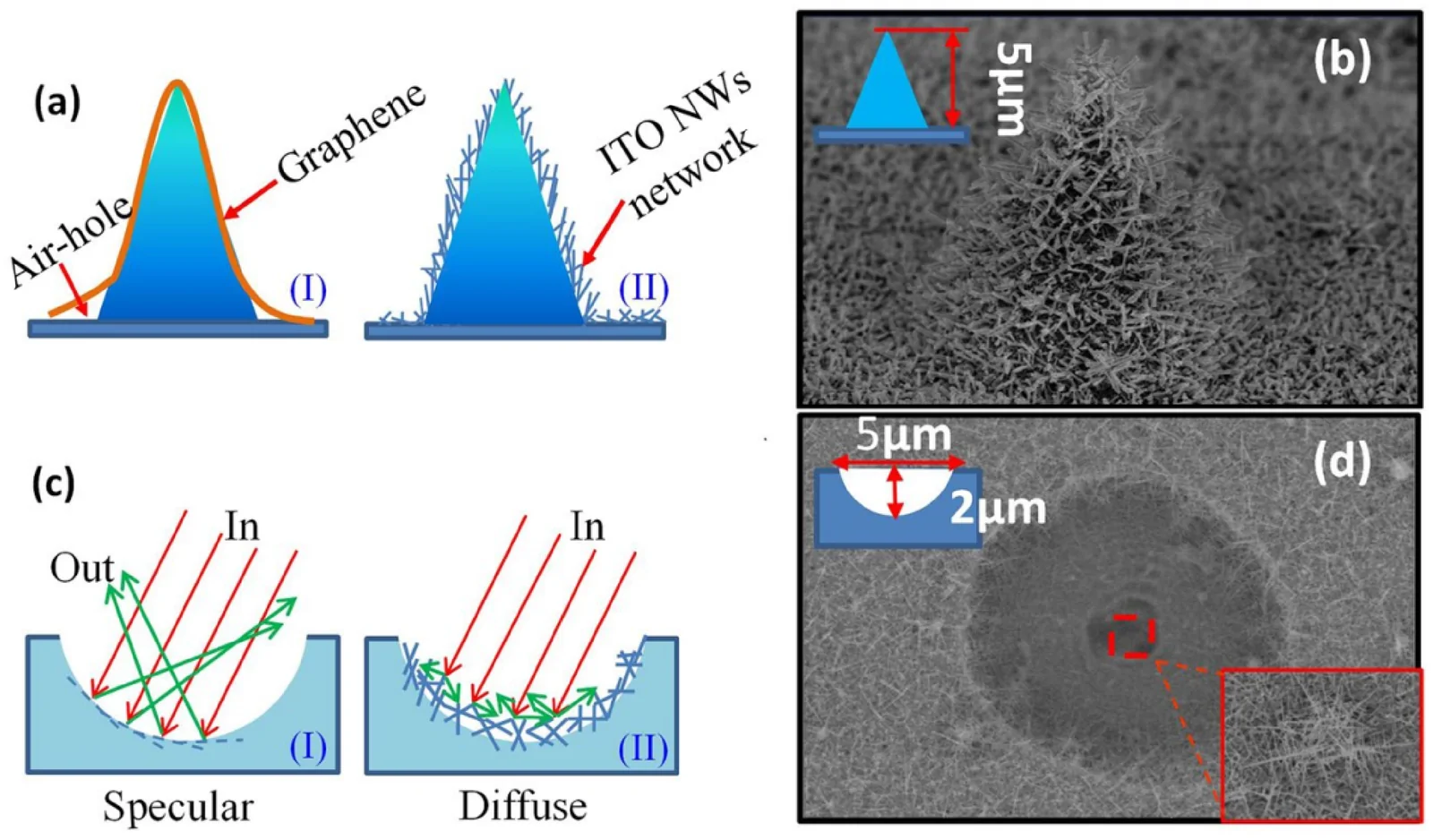
Schematic diagram of nanowire growth on (**a**) pyramid and (**c**) micro-pit substrates. The SEM image of ITO NW networks on (**b**) pyramid and (**d**) micro-pit.

Solution deposition of ITO NWs to realize transparent and flexible ITO electrodes has been demonstrated. ITO sol is deposited on a panel for antistatic or electromagnetic shielding, but its practical use is limited by suboptimal performance. In particular, the resistance of such electrodes is much higher than those of PVD-deposited ITO films. To resolve those issues, further improvements to the synthesis, dispersion, and deposition procedures for ITO NWs are still required. According to the results reported by Q. Li et al. [64], PS-assisted ITO NW networks can be grown on different morphological substrates (Figure 10), demonstrating superior optoelectronic performances with a sheet resistance of 193 Ω/sq at 96% transmission; this can greatly expand the applications of ITO NWs. Recently, breakthroughs have been made in this field, and we have reason to believe that ITO NWs will have greater applications in flexible transparent electrodes [65,66].

With the current rapid development of computational memory, more and more new materials are gradually replacing traditional memory materials, and the excellent characteristics of ITO will be used for transparent memory [67]. Fully transparent resistive random-access memory (RRAM) devices based on an ITO/X/ITO sandwiched structure have been fabricated using RF magnetron sputtering techniques [68,69,70]. ITO NWs can also be used in quantum memory. G. D. Fuchs demonstrated that a resistor terminated with a 10 µm wide ITO NWs could be used to investigate nitrogen-vacancy (NV) nuclear spins [71].

With the demand for artificial olfactory sensation, sensors incorporating ITO NWs will be popularized because of good gas-sensitivity mechanisms relating to ethanol and NO_2_. In reports of ethanol gas sensing, both response and recovery time (ms) were very fast [72,73,74]. Regarding NO_2_ sensing, ultra-low concentrations (ppb) of NO_2_ can be measured with high sensitivity [37]. The research work of X. Y. Xue further confirmed that ITO NWs can be fabricated as gas sensors for industrial applications [61].

Due to the rapid development of small electronic devices, there is a high demand for miniaturized power supplies. ITO NWs can be applied in supercapacitors [75,76,77,78,79,80]. A novel mesoporous α-Co(OH)_2_/ITO NWs heterostructure and a novel mesoporous MnO_2_/ITO NWs nanocomposite have been successfully synthesized for electrochemical energy storage application [51,52,53]. ITO NWs can not only play an important role in capacitance, but they can also be applied to solar cells [81,82,83,84,85,86,87,88]. Hong-Wen Wang used ITO NWs as 3D electrodes in dye-sensitized solar cell (DSSCs), thereby improving photoelectric conversion efficiency [58]. ITO NWs also have huge potential for improving the performance of DUV photovoltaic products [89]. ITO nanostructures have highly efficient of current injection and current spreading, while nanovoids provide a low refractive index. This can improve the optical performances of optoelectronic devices, particularly light emitting diodes (LEDs) [90,91,92,93,94].

Next, we focus on the applications of ITO nanowires in LEDs, gas sensors, resistance switches, supercapacitors, photodetectors, and solar cells.

### 3.1. Light Emitting Devices

ITO NWs with lateral branches are interwoven together to form a network film, which has good electrical conductivity and optical transmittance and can be used as ohmic electrode for LEDs. C. O’Dwyer et al. grew branched ITO nanowires on corresponding n–i–p^+^ Si/SiGe multiple-quantum-well (MQW) device structures using a molecular beam epitaxy (MBE) system; these showed good electrical (conductivity: 1053.5 ± 42.3 S cm^−1^) and optical properties (transmission: >90% in 400–1600 nm) [44]. According to Chul Huh et al.’s report [11], ITO nanowires can also enhance the charge carrier mobility and current spreading property of Si nanocrystal (NC) LEDs, significantly improving light output power by 45% and wall-plug efficiency by 38%. The typical series resistance for Si NC LEDs with ITO NWs was significantly decreased compared to those without ITO NWs, which contributed to an enhancement in the current spreading property and a decrease in the dynamic resistance. The ITO NWs increased the angle of photon escape and enhanced the light extraction efficiency (LEE).

**Figure 11:**
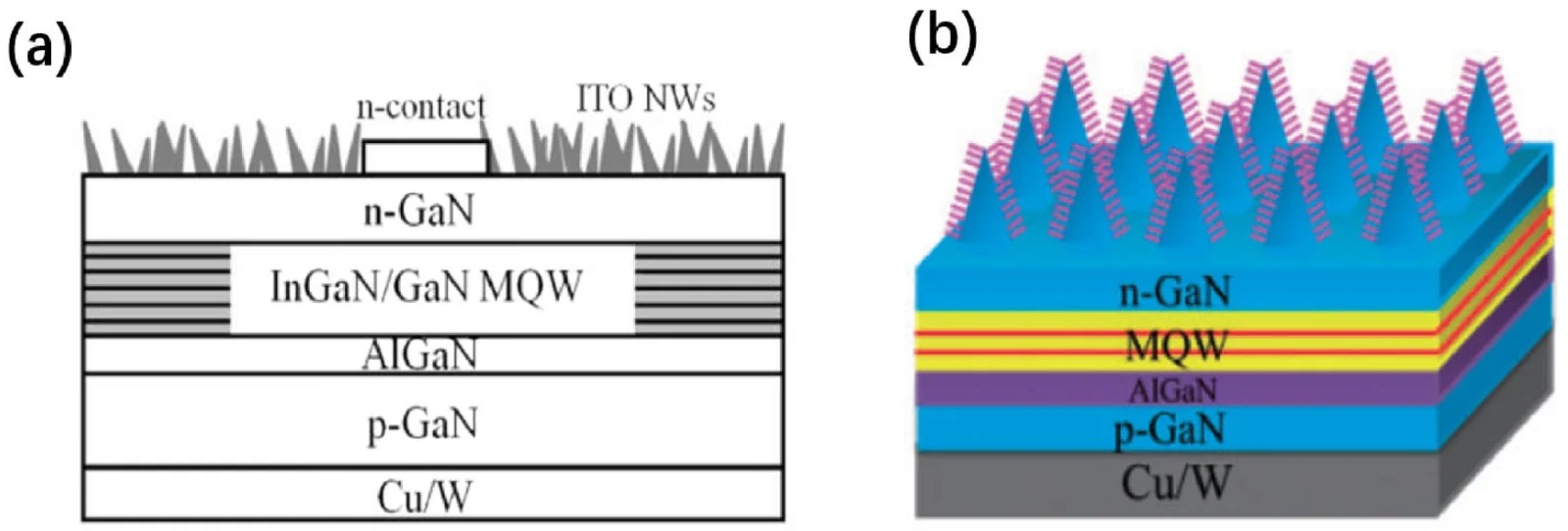
Structure diagram of ITO NWs application in VLEDs. (**a**) Blue LEDs, (**b**) green LEDs.

Zhina Gong and Qiang Li, et al. applied the ITO NWs to vertical-structure LEDs (Figure 11). The light output power for a blue LED with wavelength of 450 nm increased by 31% at a current of 150 mA compared to one without ITO NWs. A green vertical LED (VLED, λ = 517 nm) with ITO NWs showed the same enhanced light output power at 200 mA. Due to the unique morphological gradient of ITO NWs, the effective refractive index (RI) shows a natural gradient from bottom to top, making it an ideal choice for enhancing light extraction in LEDs. The power enhancement is attributed to the surface scattering and gradient RI of ITO NWs layer [93]. ITO NWs have been significantly applied in LEDs. With the continuous innovation of material design and preparation technology, the application of ITO nanostructures in LEDs will achieve greater breakthroughs in performance improvement and cost reduction, especially in the fields of micro-LED arrays and LED displays [95,96].

### 3.2. Gas Sensors

ITO nanowires are widely used as gas sensors. Due to ITO nanowires having a higher surface-to-volume ratio, deeper depletion layer, and lower power consumption than the transversal dimensions of ITO, they react more sensitively with vapor molecules. For example, at 200 ppm ethanol exposure, the sensitivity of an ITO nanowire (79.6) gas sensor was significantly higher than that of an ITO thin film (55.6) gas sensor [72]. Not only traditional ITO nanowire layers but also single or dual nanowire fabricated by laser writing can be applied to detect gas. The sensitive and fast response of ITO nanowire gas sensors, with response times within several seconds, make it possible for them to be widely applied in industrial production.

X. Y. Xue et al. synthesized ITO nanowires via thermal evaporation of In_2_O_3_, SnO, and graphite mixture powders [61]. The mechanism of the ITO nanowires used as gas sensors was as follows. When the sensors were put in an atmosphere containing ethanol gas, ethanol molecules reacted with the oxygen ions (O^−^, O^2−^, or O_2_^−^) and the electrons were released back into the conduction band of the nanowires, so that the conductivity increased. Thus, the concentration of ethanol was related to the resistance of ITO nanowire gas sensor. Usually, we define the sensitivity (S) as R_a_/R_g_, where R_a_ is the resistance in air, and R_g_ is the resistance in gas.

**Figure 12:**
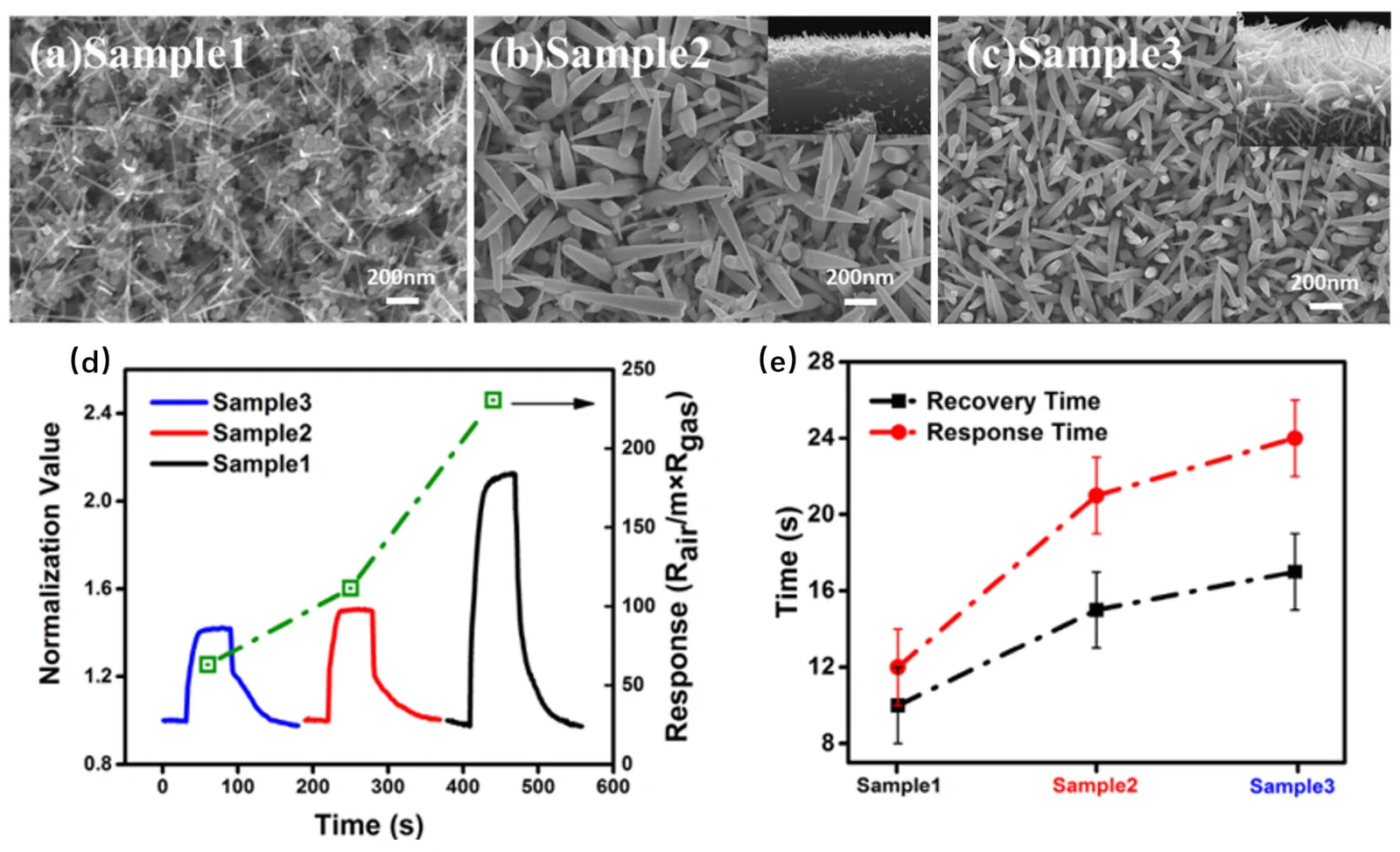
(**a**–**c**) SEM images of ITO NWs with different densities; (**d**) gas sensitivity and (**e**) response characteristics.

Many researchers have conducted in-depth research on the effect of ITO NWs on alcohol gas. For example, Q. Li et al. systematically analyzed the sensitivity of ITO NWs to ethanol of different concentrations [72]. The effect of ITO-nanowire density on the gas sensing performance was discussed. Gas sensors based on different densities of ITO nanowires have been fabricated, and the SEM images are shown in the Figure 12a–c. As shown in Figure 12d,e, the gas sensing properties reduced with the increased density. That study confirmed that selecting the appropriate nanowire density was also an important factor in high-performance gas sensors.

As well as detecting reducing gas like ethanol and methanol, ITO nanowire gas sensors also show an excellent performance on oxidizing gases like NO_2_ and ozone. M. Afshar et al. fabricated single ITO nanowires by a top-down method, as discussed in Section 2, where a single NW with well-defined position and geometry was directly nanofabricated by DC sputtering, lift-off, laser writing, and wet etching. The sensor exhibited strong gas sensitivity to NO_2_, the detection limit of which was less than 1 ppm. ITO nanomaterial is an important material system for preparing gas sensors, and research work has been ongoing. In recent years, researchers have significantly improved gas sensing properties through nanostructure modulation, surface modification, and composite material design [97,98,99,100]. In the future, flexibility, low power consumption, and intelligence will be the development direction of ITO-based gas sensors.

### 3.3. Resistive Switching

**Figure 13:**
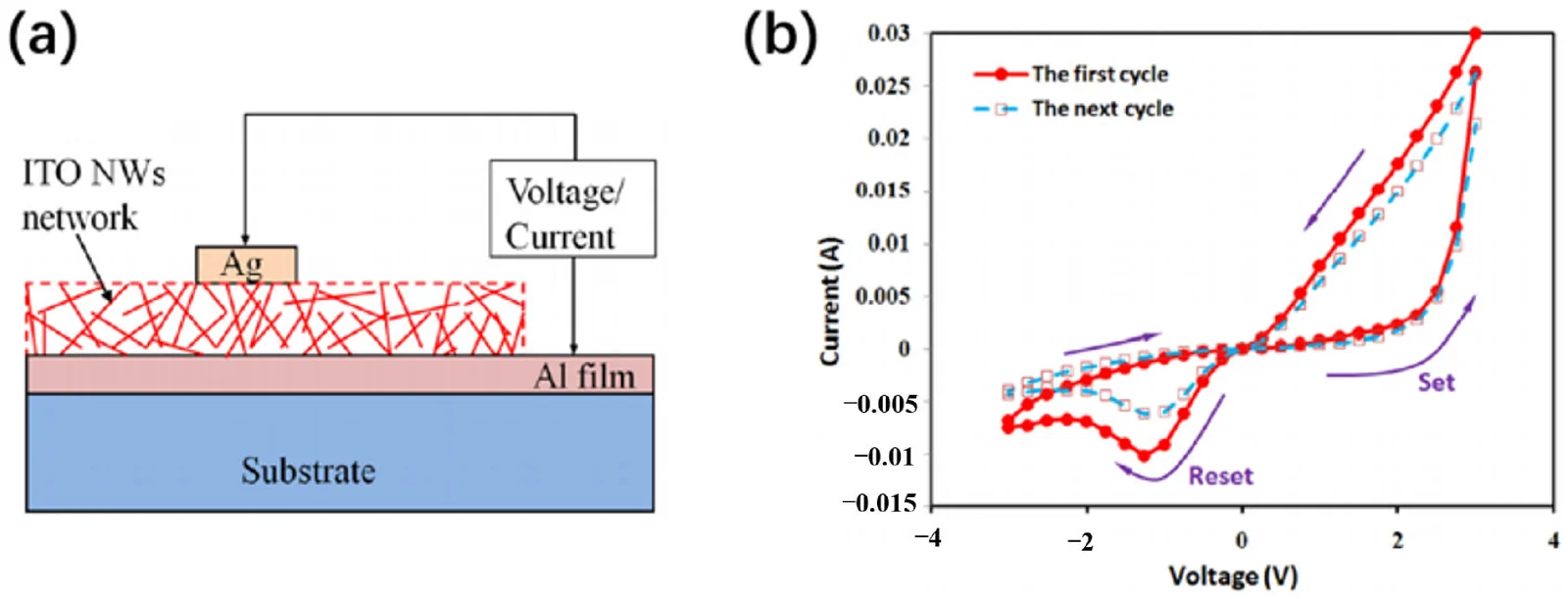
(**a**) Schematic diagram of resistive switching with ITO NWs. (**b**) Bipolar resistive switching behavior in the Ag/ITO-NWs/Al structure.

ITO-NW networks can also be used as a new metal oxide material with resistive switching behaviors. Qiang Li et al. fabricated ITO NW networks on aluminum (Al) electrodes. The bipolar resistive switching behaviors of ITO NW networks were first observed in the Ag/ITO-NWs/Al structure. (Figure 13) Then, a novel flexible transparent (FT) resistive random access memory (ReRAM) device using Ag/ITO-NW networks on a poly(dimethylsiloxane) (PDMS) substrate was reported [54,55]. That study confirmed the reversible and stable bistable resistance switching behavior of ITO NWs, which provided potential for the application of non-volatile memory in flexible transparent devices. At present, the most commonly used method is to combine ITO with other materials to prepare resistance switches [101,102].

### 3.4. Supercapacitors

Q. Li et al. prepared ITO NWs on Ni foam by magnetron sputtering as the active electrode of a pseudocapacitor. A self-stabilizing integrated electrode was designed and manufactured by combining ITO NWs. The voltage stabilization time during the discharge process of supercapacitors almost doubled, revealing the potential application of integrated electrodes as voltage self-stabilizing supercapacitors [75]. According to Duc Tai Dam et al.’s work, ITO NWs can also be used electrodes in supercapacitors with unique mesoporous α-Co(OH)_2_/ITO NWs heterostructures. At a current density of 10 A/g, the electrode kept 90.9% of its initial specific capacitance after 500 cycles, which indicatied that the prepared electrode had long-term electrochemical stability [52]. Mesoporous MnO_2_/ITO NWs nanocomposite dramatically improved the accessibility of electrolytes and reduced the electron transport distance. The combination of high conductivity of ITO NWs and the high surface area of mesoporous MnO_2_ can be considered to represent a promising electrode-fabricating technique [51,53]. Jingwei Du et al. also reported an ITO NWs 3D network that served as the current collector and 3D scaffold for a micro supercapacitor (MSC). In related research, 3D electrodes should be designed to offer more growing sites for active materials. ITO NWs have excellent conductivity and good adhesion to substrates without the need for introducing other conductive layers or adhesives, because of their vertically arranged nanostructures.

### 3.5. Photodetectors

UV sensing photodetectors provide unique performance in light communications, missile-tracking, biolabeling, weather monitoring, and flame detection [103,104,105,106,107]. ITO NWs exhibit transmittance of more than 80%, enhanced charge collection, extremely low resistivity in the order of 10^−4^ Ω cm, and high electron concentration of 2.6 × 10^20^ cm^−3^. Hyunki Kim et al. fabricated a UV photodetector based on an ITO NWs network [60], which resulted in better light trapping, elevated carrier collection efficiency, suppressed reflection, and reduced recovery time in photo response. By applying a reverse bias voltage, it improved the current-time (I-t) response characteristics of the NiO/ZnO/ITO NW UV detector under 365 nm light with a light intensity of 3 mW cm^−2^. The sensitivity [(I_light_ − I_dark_)/I_dark_] was 11.71, which was much higher than 0.42 without this structure. The response time and recovery time were 7.05 ms and 17.61 ms, respectively. These results were significantly shorter than those for the detector without ITO NWs in the authors’ previous report. Therefore, NiO/ZnO UV detectors assisted by ITO NWs can become promising candidates to detect even weak UV signals in numerous fields.

### 3.6. Solar Cells

Q. Li et al. prepared ITO nanowires on a Si surface with a micro hole array to form a super-broadband antireflection layer for solar cells. The ITO NW network was not only a refractive index gradient film, but also a surface roughened layer, which could be used as a super-broadband antireflection layer. ITO NWs were used in μ-hole array, which greatly reduced the reflection of 400–1200 nm band lights. When the light entered the μ-holes covered with ITO NWs, the reflected light was completely confined in the μ-holes after multiple scattering [81]. ITO NW arrays can also be applied into the construction by coating TiO_2_ layers in the 3D electrodes for solar cells, as in Hong-Wen Wang et al.’s work [89]. By comparing the photovoltaic conversion efficiency of solar cells using planar ITO electrodes and ITO NW array electrodes, that study found a 4.3% improvement in photovoltaic conversion efficiency in dye-sensitized solar cells (DSSCs) made with ITO NWs. The use of ITO NW arrays as a 3D electrode for DSSCs has been proven to be a feasible method for improving photovoltaic performance, especially in further optimizing synthesis routes and cell structures. At present, the combination of ITO NWs with new photovoltaic materials (such as perovskite) has effectively improved the conversion efficiency of solar cells, reaching 30.72% with a fill factor of 88.99% [83,84].

## 4. Conclusions and Perspectives

This paper provides an overview of the growth and device applications of ITO NWs, serving as a comprehensive reference for a thorough understanding of ITO NWs. We systematically summarize the process parameters and nanowire morphologies for preparing ITO NWs using methods such as electron beam deposition, pulsed laser deposition, DC/RF sputtering, thermal treatment, and laser etching. The temperatures of different growth methods range from 150 °C to 930 °C, and the diameter of ITO NWs can vary from 20 nm to 700 nm. A single nanowire can reach up to 200 μm in length. Additionally, we review the applications and performance enhancements of ITO NWs in devices such as LEDs, gas sensors, resistive switching, supercapacitors, photodetectors, and solar cells. Meanwhile, some cutting-edge research is also underway, including into the nonlinear effects of ITO NWs and precise control of nanowire morphology.

In the future, ITO NWs will continue to be combined with other materials to enhance device performance [108,109,110,111,112,113,114,115,116,117,118], especially in the following three areas. First, in the field of flexible devices, flexible electronics technology not only integrates technologies from fields such as electronic circuits, functional materials, and micro-nano fabrication, but also spans industries including semiconductors, packaging, testing, materials, chemical engineering, printed circuits, and display panels. It is poised to drive a CNY trillion market, assist traditional industries in enhancing their added value, and bring revolutionary changes to industrial structures and human life. Leveraging the excellent transparency and flexibility of ITO NWs, they will further play a role in the field of wearable, bendable, flexible, and invisible electronic products. Notably, ITO NWs have certain development potential for high-speed, high-density, high-transparency flexible memory, and oxide nanowire-based high-performance transistors. Second, supercapacitors, with their extremely fast response time and ultra-long cycle life, are key components in the construction of a new hybrid energy storage system, and their market value is expected to exceed USD ten billion in the next five years. ITO NWs can be applied in the field of light-charging supercapacitors. These devices are suitable for scenarios such as wearable electronics, aerospace equipment, and agricultural environmental monitoring nodes. Combined with AI and IoT technologies, they can create fully self-powered systems, representing an important direction for future green energy storage development. Third, photovoltaic technology, capable of directly converting solar energy into electrical energy, stands as the most stable and efficient new energy technology currently available. It significantly reduces humanity’s reliance on fossil fuels, cuts down on greenhouse gas emissions, and aids in the realization of global “dual-carbon” goals. Within the solar photovoltaic industry, the integration of ITO NWs with perovskite, a novel solar cell material, not only enhances the photoelectric conversion efficiency of devices but also holds immense potential for future applications.

In conclusion, we believe that ITO NWs, as an important form of ITO materials, will have a greater impact on the optoelectronic device market in the near future, enhancing product performance and serving smart devices.

## Figures and Tables

**Table 1:** Summary of ITO NW characteristics.

Methods	Temp.	Diameter	Length	Resistance	Trans. (T%)	With/Wo Branches	Ref.
Glancing angle deposition (1)	200–260 °C	/	0.5–1 μm	3300 ± 400 Ω·cm	/	with	[38]
Nd:YAG pulsed laser deposition	~250 °C	20–30 nm	700–800 nm	/	/	with	[23]
Ion beam assisted deposition	<150 °C	/	/	~115 Ω	/	with	[10]
Magnetron sputtering	>175 °C	20–200 nm	1–10 μm	/	/	w/o	[24]
Thermal evaporation-deposition	~600 °C	120 nm	1.75 μm	6.11 × 10^−4^ Ω·cm	>75%	w/o	[1]
DC sputtering (1)	350 °C	∼50–100 nm	3~5 μm	8 Ω/sq	/	with	[3]
Pulsed laser deposition (1)	500 °C	60 nm	2.5 μm	/	/	with	[43]
Pulsed laser deposition (2)	250 °C	∼20 nm	∼700–800 nm	1.6 × 10^−4^ Ω·cm	∼90–96%	with	[19]
Excimer laser ablation	500 °C	70–100 nm	4.5 μm	/	/	with	[44]
Molecular beam epitaxial deposition	300–650 °C	10–15 nm.	40–500 nm	1–4 × 10^−4^ Ω·cm	~80–90%	with	[45]
Electron-beam evaporation (1)	150–400 °C	10–40 nm	0.1~4 μm	0.71–1.5 kΩ/sq	~70–85%	with	[6]
Electron-beam deposition via PS	300 °C	/	/	~200 Ω/sq	>90%	with	[20]
Electron-beam evaporation (2)	400 °C	20–50 nm	0.8–1.2 μm	/	/	with	[8]
Electron-beam evaporation (3)	300 °C	/	/	~115 Ω	/	with	[10]
Low-pressure chemical vapor deposition (LPCVD)	800 °C	50–100 nm	100 μm	/	/	w/o	[34]
Thermal CVD	540 °C	70 nm	>10 μm	>10^−3^ Ω	/	w/o	[46]
Glancing angle deposition (2)	375–500 °C	23 ± 1–56 ± 3 nm	/	81.2 Ω/sq	90.8%	with	[5]
Vapor transport method based on Yttrium-stabilized zirconia (YSZ, 1)	800–900 °C	50 ± 9–140 ± 18 nm	335–980 ± 140 nm	/	/	w/o	[2]
Vapor transport method based on Yttrium-stabilized zirconia (YSZ, 2)	800–900 °C	100 nm	/	/	/	with	[16]
Co-precipitation annealing	500 °C	50–70 nm	10 μm	/	/	w/o	[30]
Vapor transport method based on Yttrium-stabilized zirconia (YSZ, 3)	900 °C	<200 nm	2.5 μm	/	/	w/o	[33]
Electron-beam evaporation (4)	400 °C	20–50 nm	0.8–1.2 µm	/	/	with	[36]
Pulsed laser deposition (3)	750 °C	/	/	10^−4^ Ω·cm	/	w/o	[47]
Thermal evaporation (1)	750 °C	99–366 nm	20 µm	/	/	with	[9]
Low-pressure chemical vapor deposition (LPCVD)	800 °C	50 nm	100 µm	/	/	w/o	[34]
Catalyst-assisted heteroepitaxial growth	800–900 °C	59 nm ± 13 nm	∼6–8 µm	/	/	with	[35]
Thermal treatment	650–750 °C	80–300 nm	20 µm	/	/	w/o	[29]
Heteroepitaxial growth	840 °C	86 nm ± 12 nm	∼2.5 µm	/	/	with	[48]
Thermal evaporation (2)	920 °C	100–200 nm	10–100 µm	/	/	w/o	[28]
Thermal evaporation (3)	~850–900 °C	100–300 nm	/	300–1500 µΩ·cm	/	w/o	[49]
Electrospinning	900 °C	80–120 nm	45.0 μm	/	/	w/o	[50]
Chemical vapor deposition (CVD, 1)	700 °C	100 nm	/	/	/	w/o	[51]
Chemical vapor deposition (CVD, 2)	400–600 °C	/	50 μm	/	/	w/o	[52]
Chemical vapor deposition (CVD, 3)	400–600 °C	/	30–50 μm	/	/	w/o	[53]
Electron beam evaporation via PS	280 °C	/	/	/	/	w/o	[54]
Electron beam evaporation (5)	300 °C	40–50 nm	∼2 μm	/	/	w/o	[55]
RF sputtering (1)	210–260 °C	30–100 nm	800 nm	/	/	with	[56]
RF sputtering (2)	500 °C	/	/	90 Ω/sq	85%	with	[57]
KrF excimer pulsed laser deposition	300–500 °C	<50 nm	/	/	/	w/o	[11]
Molecular beam epitaxy	300–650 °C	8–20 nm	40–500 nm	1 × 10^−4^–4 × 10^−4^ Ω·cm	~90%	with	[45]
Nd:YAG pulsed laser deposition	250 °C	<50 nm	∼700–800 nm	1.6 × 10^−4^ Ω·cm	∼90–96%	with	[19]
Electrophoretic deposition	500 °C	100–200 nm	10 μm	/	/	w/o	[58]
Hydrogen thermal reduction vapor transport	600 °C	40–200 nm	/	/18	/	w/o	[59]
DC sputtering (2)	600 °C	/	/	18 Ω/sq	~80%	w/o	[60]
Laser beam writing	/	350–700 nm	90–200 μm	3.5 × 10^−4^ Ω·cm	/	w/o	[37]
Thermal evaporation (4)	930 °C	70–150 nm	>10 μm	/	/	w/o	[61]

## Data Availability

No new data were created or analyzed in this study. Data sharing is not applicable to this article.
